# *Candida auris* Infection Leading to Nosocomial Transmission, Israel, 2017

**DOI:** 10.3201/eid2404.171715

**Published:** 2018-04

**Authors:** Ana Belkin, Zeala Gazit, Nathan Keller, Ronen Ben-Ami, Anat Wieder-Finesod, Ana Novikov, Galia Rahav, Tal Brosh-Nissimov

**Affiliations:** Sheba Medical Center, Tel Hashomer, Israel (A. Belkin, Z. Gazit, N. Keller, A. Wieder-Finesod, G. Rahav, T. Brosh-Nissimov);; Sackler Medical School, Tel Aviv University, Tel Aviv, Israel (A. Belkin, R. Ben-Ami, A. Wieder-Finesod, G. Rahav, T. Brosh-Nissimov);; Ariel University, Ariel, Israel (N. Keller);; Tel Aviv Sourasky Medical Center, Tel Aviv (R. Ben-Ami, A. Novikov)

**Keywords:** *Candida auris*, travel, phylogeography, nosocomial infection, outbreak, fungi, South Africa, Israel

## Abstract

A patient transferred from South Africa to Israel acquired a *Candida auris* infection. Phylogenetic analysis showed resemblance of *C. auris* to isolates from South Africa but not Israel, suggesting travel-associated infection. *C. auris* infection occurred weeks later in another patient at the same hospital, suggesting prolonged environmental persistence.

*Candida auris* is a multidrug-resistant yeast that has emerged over the past 3 years to cause nosocomial outbreaks in multiple countries. *C. auris* can cause serious invasive infections, may spread between patients, and can survive for months on hospital room surfaces (*1*). Whole-genome sequencing has determined the presence of country-specific clades, which differ from one another by thousands of single-nucleotide polymorphisms (*2*). The mode of spread between countries remains unclear. We present a case of international *C. auris* transmission related to a medically repatriated patient.

A previously healthy 25-year-old Israeli man (patient A) was admitted to a hospital in Johannesburg, South Africa, after a motor vehicle accident on July 24, 2016. He had severe limb injury and underwent open reduction and internal fixation on both femurs, complicated by fat emboli, acute respiratory distress syndrome requiring mechanical ventilation, and ventilator-associated pneumonia. He was empirically treated with broad-spectrum antimicrobial drugs and caspofungin. Three weeks after the accident, he was transferred to the intensive care unit (ICU) of Sheba Medical Center, Tel Hashomer, Israel. Ten days after his arrival, a deep surgical-site infection developed in his left thigh. We initiated debridement and broad-spectrum antimicrobial drugs. After cultures obtained during surgery grew extended-spectrum β-lactamase–producing *Klebsiella pneumoniae* and meropenem-resistant *Pseudomonas aeruginosa,* we initiated contact isolation. Two of 3 deep-wound cultures grew *C. auris*. Two days later, 1 blood culture grew *C. parapsilosis*. We administered amphotericin B and appropriate antibacterial drugs, discontinuing amphotericin B after 10 days due to increased creatinine. The surgical site healed, and the patient was transferred to a rehabilitation unit. Rectal and skin surveillance cultures obtained 4 weeks after the first isolation of *Candida* were negative for *C. auris*. Routine ICU environmental disinfection included daily bleach cleaning of surfaces and quaternary ammonium wipes of sensitive medical equipment.

In January 2017, we isolated *C. auris* from a urine culture obtained through a catheter of a 70-year-old patient (patient B) who was admitted to the Sheba Medical Center ICU 6 weeks after the discharge of patient A. Patient B had not traveled abroad recently. Surveillance cultures (urine, axilla, perineum) of patients in the ICU at the time of *C. auris* isolation of either patient A or B were negative for *C. auris*. One environmental sample from the floor next to patient B’s bed in proximity to the urinary catheter bag was positive for *C. auris*. All other environmental samples were negative. We removed the urinary catheter without further antimicrobial therapy. Strict environmental cleaning was performed in the ICU.

We performed drug susceptibility testing using broth microdilution in accordance with Clinical Laboratory Standards Institute methods (https://clsi.org/standards/products/microbiology/documents/m27/) and reported results with preliminary breakpoints as published by the US Centers for Disease Control and Prevention (*3*). The study was approved by the Sheba Medical Center institutional review board.

We identified isolates as *C.* *auris* by matrix-assisted laser desorption/ionization time-of-flight mass spectrometry (Bruker Daltonik, Bremen, Germany) and as *C*. *parapsilosis* by the Phoenix system (Becton Dickinson, Franklin Lakes, NJ, USA). Sequence alignment with *C. auris* type strain CBS10913T produced similarity scores of 98% for internal transcribed spacer and 100% for large subunit ribosomal DNA segments for all 4 strains. Internal transcribed spacer and large subunit sequences of isolates from both patients were 100% identical to strains for MRL293 and MRL208 from South Africa (*4*) and distinct from sequences of strains previously isolated in our hospital and in Tel Aviv (Figure) (*5*).

**Figure:**
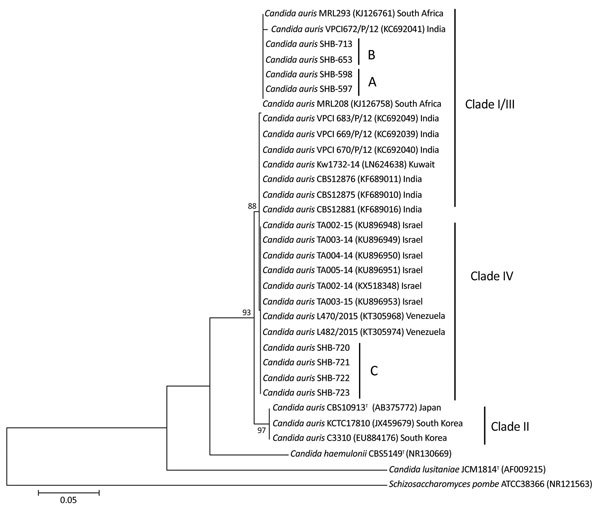
Phylogenetic analysis of *Candida auris* strains from 2 patients in Israel. Tree was generated using the neighbor-joining method. Internal transcribed spacer sequences of *C. auris* strains were aligned with the *C. auris* type strain CBS5149^T^, strains previously isolated in Tel Aviv (TA002-TA005), and additional clinical strains available from GenBank. A indicates isolates from patient A, who was transferred from South Africa to Sheba Medical Center in Israel in late 2016. B indicates isolates from patient B, who was admitted to the same unit 6 weeks after the discharge of patient A, in January 2017; SHB-713 is an environmental sample from the floor near patient B’s bed. C indicates isolates from sputum and urine from 2 different patients infected with *C. auris* in Sheba Medical Centerl during 2017 (SHB-720–723). The percentages of replicate trees in which the associated taxa clustered together in the bootstrap test (500 replicates) are shown next to the branches. GenBank accession numbers are given in parentheses, and countries of origin are listed. *C. lusitaniae* JCM1814^T^ and *Schizosaccharomyces pombe* ATCC38366 were used as outgroups. Scale bar indicates nucleotide substitutions per site.

*C. auris* isolates from patients A and B were resistant to fluconazole and susceptible to anidulafungin and had high MICs to voriconazole (>8 μg/mL). One isolate was resistant to amphotericin B (MIC 2 μg/mL) (*3*), although a recent study suggested a higher epidemiologic cutoff that defines the isolate as susceptible (*6*).

Nosocomial outbreaks associated with *C. auris* were reported from several countries and continents including India, South Africa, Venezuela, Pakistan, and the United States (*2*,*7*,*8*). Sporadic cases were reported from Israel (*5*). Echinocandin exposure, which preceded *C. auris* infection in patient A, was also reported in South Africa (*2*). Environmental contamination appears to be a common mode of *C. auris* spread within medical facilities (*1*); it is the suspected cause for the 2 cases reported here, despite the time between them. The use of quaternary ammonium compounds, which are less effective than bleach, for disinfecting equipment might contribute to persistence of *Candida* (*9*). 

International travel is an increasingly recognized risk factor for infection with drug-resistant pathogens. Our investigation underscores the potential role of international travel and especially the transportation of patients between countries as a mode of *C. auris* dissemination. The wide genetic gap between country-specific clades allows the use of ribosomal DNA typing as a tool for identifying the geographic origin of specific isolates (*2*,*5*). A similar approach was used to demonstrate multiple transmission events into the United Kingdom (*10*).
